# Combined prognostic value of the SUVmax derived from FDG-PET and the lymphocyte-monocyte ratio in patients with stage IIIB-IV non-small cell lung cancer receiving chemotherapy

**DOI:** 10.1186/s12885-021-07784-x

**Published:** 2021-01-14

**Authors:** Kewei Zhao, Chunsheng Wang, Fang Shi, Yong Huang, Li Ma, Minghuan Li, Yipeng Song

**Affiliations:** 1 Department of Radiation Oncology, Yantai Yuhuangding Hospital, 20 Yudong Road, Yantai, 264000 Shandong People’s Republic of China; 2 Department of Radiation Oncology, Shandong Cancer Hospital and Institute, Shandong University, 440 Jiyan Road, Jinan, 250117 Shandong People’s Republic of China; 3 Department of Nuclear Medicine, Shandong Cancer Hospital and Institute, Shandong University, 440 Jiyan Road, Jinan, 250117 Shandong People’s Republic of China

**Keywords:** Non-small cell lung cancer, Maximum standardized uptake value, Lymphocyte-monocyte ratio, Response, Survival

## Abstract

**Background:**

We evaluated the prognostic potential of tumor ^18^F-fluorodeoxyglucose (FDG) uptake derived from positron emission tomography (PET) and known inflammatory hematological markers, both individually and in combination, for chemosensitivity and survival in patients with stage IIIB-IV non-small cell lung cancer (NSCLC) receiving first-line chemotherapy.

**Methods:**

A total of 149 patients with stage IIIB and IV NSCLC (based on TNM 7th edition) were retrospectively reviewed. Maximum standardized uptake value (SUVmax) were used to quantitatively assess FDG uptake. The lymphocyte-monocyte ratio (LMR), neutrophil-lymphocyte ratio (NLR) and platelet-lymphocyte ratio (PLR) were selected as hematological markers. Receiver operating characteristic (ROC) curves were constructed for the determination of optimal cut-off values to predict chemotherapeutic response.

**Results:**

Patients with SUVmax > 11.6 or LMR ≤3.73 exhibited a significantly lower objective response rate (ORR) to chemotherapy (*p* < 0.001 and *p* < 0.001). Through multivariable logistic regression analysis, both the SUVmax and LMR were identified as independent predictive factors for chemotherapeutic response (*p* = 0.001 and *p* < 0.001). Furthermore, a multivariable Cox proportional hazard model identified a high SUVmax (> 11.6) and low LMR (≤3.73) as independent predictors of poor PFS (*p* < 0.001 and *p* = 0.025) and OS (*p* < 0.001 and *p* = 0.032). A novel score system was constructed based on the SUVmax and LMR (SUV_LMR score), and patients were stratified into three subgroups. The patients with a score of 0 had a significantly higher ORR (88.9%) than did those with a score of 1 (59.6%) and score of 2 (25.0%) (*p* < 0.001). Moreover, multivariable Cox analysis further identified the SUV_LMR score as an independent prognostic factor for PFS (*p* < 0.001) and OS (*p* < 0.001).

**Conclusions:**

Pre-treatment SUVmax and LMR were not only predictive factors for chemotherapeutic response but also independent prognostic factors of survival in stage IIIB-IV NSCLC. Moreover, the SUV_LMR score, which is based on primary tumor metabolic activity and the systemic inflammatory response, might provide a promising tool to predict chemosensitivity, recurrence and survival of advanced NSCLC.

## Background

Globally, lung cancer has 5-year survival rates as low as 15% [1]. Non-small cell lung cancer (NSCLC) is the most common pathological type of lung cancer, accounting for 85% of all cases [1]. Owing to lack of early symptoms, close to 70% of patients are already in late stage when they are initially diagnosed with NSCLC, and most of them have lost the opportunity for surgical therapy [2]. For advanced stage IIIB and IV NSCLC, the front line therapy remains platinum-based doublet chemotherapy [3]. However, the response rate to chemotherapy and the survival in advanced NSCLC are not ideal [4]. In order to develop appropriate treatment guidelines and balance therapeutic toxicity in individual patients, precise prediction of chemotherapy sensitivity and prognosis is crucial. The anatomical range of the tumor, which is expressed by the tumor, node, metastasis (TNM) classification, is recognized as the most powerful prognostic factor for lung cancer [5]. However, TNM staging is only an anatomical description of the tumor and is not enough to develop treatment programs and evaluate prognosis. In the future, the most promising will be the composite prognostic model for NSCLC based on biological characteristics in conjunction TNM staging [6].

Reprogramming energy metabolism is one of the biological hallmarks in cancer [7]. Indeed, significantly elevated glucose uptake and utilization have been observed in various types of tumors [7]. ^18^F-fluorodeoxyglucose positron emission tomography/computed tomography (^18^F-FDG PET/CT), which can noninvasively quantify glucose uptake that precedes anatomic changes, has been widely applied in NSCLC diagnosis, staging, response evaluation and survival prediction [8]. Maximum standardized uptake value (SUVmax) is a commonly used parameter for quantifying FDG uptake and has been reported to have a strong prognostic value for NSCLC [9–13]. The common conclusion of these studies is that higher values of SUVmax could predict a high risk of disease recurrence or death. However, this conclusion is still controversial since some studies also demonstrated that SUVmax cannot provide reliable prognostic information for NSCLC patients [14, 15]. Therefore, the prognostic role of SUVmax in NSCLC needs further validation.

Tumor-promoting inflammation is another recognized biological hallmark of cancer [7]. Accumulating evidence has demonstrated that the cancer-related inflammation response can enhance tumor progression by facilitating angiogenesis, invasion, and metastasis [16–18]. Hematological markers of systemic inflammation include the lymphocyte-monocyte ratio (LMR), neutrophil-lymphocyte ratio (NLR) and platelet-lymphocyte ratio (PLR), all of which are prognostic markers for a range of solid tumors, including NSCLC [19–26]. However, the optimal hematological markers for predicting clinical outcomes in NSCLC have yet to be defined [22–26].

The combined evaluation of FDG uptake of primary tumor and systemic inflammatory response may provide complementary information and may be highly effective at predicting outcomes in advanced NSCLC patients. Hence, we combined the two factors to explore their integrated predictive value for treatment response and survival in stage IIIB-IV NSCLC patients.

## Methods

### Patient selection

We reviewed the clinical data of patients pathologically diagnosed with NSCLC from September 2013 to June 2017. All enrolled patients received ^18^F-FDG PET/CT scanning within two weeks prior to treatment. According to the PET/CT and other imaging examinations, all patients were staged based on 7th TNM staging system. Patients were included according to: 1) age, 18–75 years; 2) clinical stage IIIB or IV; 3) Eastern Cooperative Oncology Group performance status (ECOG PS) 0–1; 4) no history of other malignancies; 5) without acute infections or autoimmune diseases; and 6) received ≥4 cycles of first-line platinum-based doublet chemotherapy. Data regarding age, sex, ECOG PS, smoking history, lesion type, histological type, and clinical stage were extracted and analysed. All patients received first-line chemotherapy and additional radiotherapy if indicated.

### ^18^F-FDG PET/CT scanning and image analysis

^18^F-FDG PET/CT was performed on an advanced PET/CT scanner (Discovery LS, GE Healthcare). All of them with 6-h fasting and ensure blood glucose level was< 200 ml/dL before receiving an average of 5.5 MBq/kg ^18^F-FDG intravenous injections. One hour later, a whole-body PET and CT scans began, ranging from the base of the skull to the proximal thigh. After CT-derived attenuation correction, the PET images were reconstructed by the ordered subsets expectation maximization algorithm. The reconstruction layer thickness was 4.25 mm and the image matrix size were 128 × 128. The CT, PET and fused images were displayed on the Xeleris workstation (GE Healthcare). Two experienced diagnostic specialists reviewed the PET/CT images independently. FDG uptake was quantified using SUV, and the highest pixel value of SUV within region of interest (ROI) was defined as the SUVmax.

### Definition of hematological markers

All patients underwent routine blood tests within one week before treatment. The peripheral counts of white blood cell (WBC), lymphocytes, neutrophils and platelets were recorded. The LMR was defined as the ratio of the lymphocyte count to the monocyte count. The NLR was calculated as the neutrophil count divided by the lymphocyte count, and the PLR was calculated as the platelet count divided by the lymphocyte count.

### Evaluation of response and follow-up

After four weeks of treatment completion, tumor response was assessed based on the RECIST1.1. According to these criteria, responders were classified as complete response (CR) and partial response (PR), and non-responders were classified as stable disease (SD) and progressive disease (PD). Objective response rates (ORRs) were calculated as the percentage of CR and PR among all treated patients.

The patients were followed up every 3 months for the first 2 years, every 6 months for the next 3 years, and every year after 5 years. Overall survival (OS) was calculated from the date of treatment initiation to the date of death or final follow-up. Progression-free survival (PFS) was calculated from the date of treatment initiation to the date of diagnosis of local recurrence/distant metastasis, or final follow-up. Medical records and telephone interviews were used to compile follow-up data of patients. The median follow-up was 16.1 months (range: 4.7–63.2 months). The last follow-up date was December 10, 2018.

### Statistical analysis

Study data were analyzed on SPSS version 22.0 and MedCalc program version 18.11. Correlations between SUVmax and hematological markers were evaluated using Spearman’s correlation coefficient tests. Receiver-operating characteristic (ROC) curves were constructed to obtain optimal cut-off values of SUVmax and hematological markers for identifying the treatment responders. Delong’s tests were used to compare the area under the curves (AUCs) of the three hematological markers. The correlation of each variables and treatment response were assessed via logistic regression models. Kaplan–Meier (KM) curves and log-rank tests were used for the assessment of patient survival. Prognostic factors with *p* < 0.05 in univariable analyses were entered into multivariable Cox proportional hazards model. All tests were two-sided and p < 0.05 was deemed statistically significant.

## Results

### Patient data

In total, 149 eligible patients were included into analysis. Among them, 111 (74.5%) were male and 38 (25.5%) were female, with a median age of 61 (range: 36–75) years. Regarding histological subtype, 55 (36.9%) had squamous cell carcinoma, and 94 (63.1%) were diagnosed as adenocarcinoma. Amongst them, 69 (46.3%) were at stage IIIB, and 80 (53.7%) were at stage IV. All included patients received 4–6 (median: 4) cycles of first-line platinum-based chemotherapy and the regimens including cisplatin/paclitaxel, cisplatin/docetaxel, cisplatin/gemcitabine or cisplatin/pemetrexed. Detailed baseline characteristics were listed in the Table 1.

**Table 1:** Baseline characteristics of 149 patients

Characteristics	Number (%)	Median (range)
Age (years)		61 (36–75)
≤ 65	95 (63.8%)	
>65	54 (36.2%)	
Sex		
Male	111 (74.5%)	
Female	38 (25.5%)	
Smoking		
Never	60 (40.3%)	
Ever	89 (59.7%)	
ECOG PS		
0	73 (49.0%)	
1	76 (51.0%)	
Histological type		
Adenocarcinoma	94 (63.1%)	
Squamous	55 (36.9%)	
Lesion type		
Central	78 (52.3%)	
Peripheral	71 (47.7%)	
Clinical stage		
IIIB	69 (46.3%)	
IV	80 (53.7%)	
CEA		
Normal	95 (63.8%)	
Increased	54 (36.2%)	
Albumin		
Decreased	50 (33.6%)	
Normal	99 (66.4%)	
WBC (× 10^9^/L)		7.6 (3.6–12)
LMR		3.25 (0.98–12.5)
NLR		2.78 (1.05–8.14)
PLR		143 (35–352)
SUVmax		11.8 (3.3–28.5)

*ECOG PS* Eastern Cooperative Oncology Group Performance Status, *CEA* carcinoembryonic antigen, *WBC* white blood cell, *LMR* lymphocyte-monocyte ratio, *NLR* neutrophil-lymphocyte ratio, *PLR* platelet-lymphocyte ratio, *SUVmax* maximum standardized uptake value

### Correlation between SUVmax and hematological markers

**Fig. 1:**
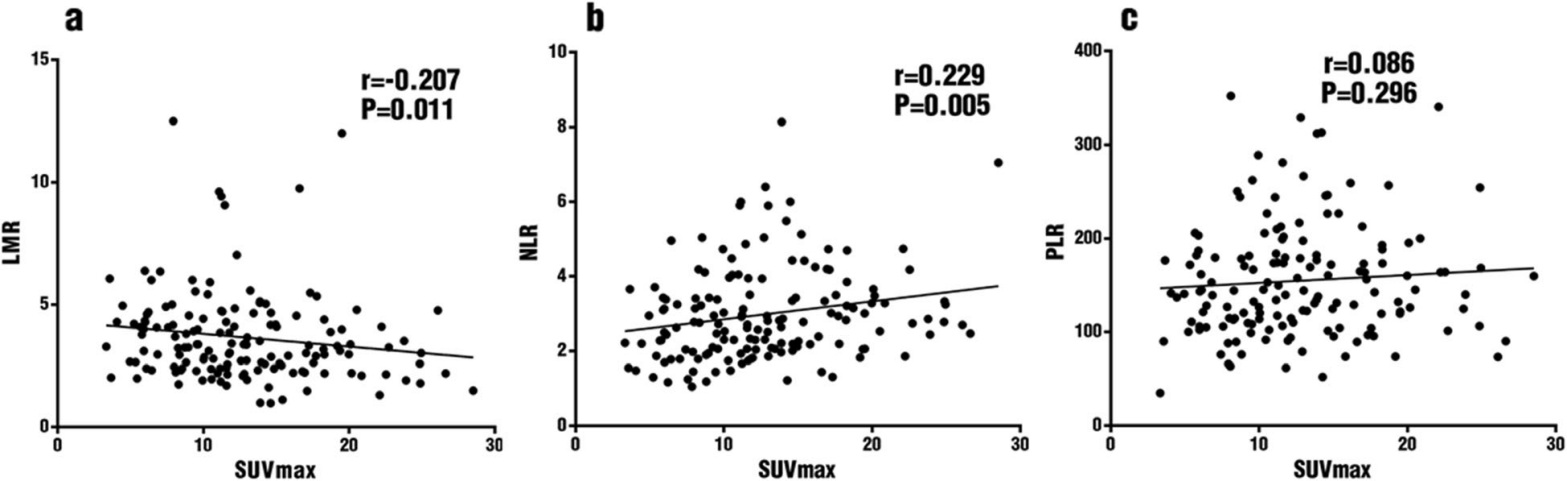
Correlation between SUVmax and hematological markers LMR **a**, NLR **b** and PLR **c**

From correlation analysis, LMR (r = − 0.207, *p* = 0.011) and NLR (r = 0.229, *p* = 0.005) showed significant yet quite weak correlations with SUVmax, while PLR did not (r = 0.086, *p* = 0.296) (Fig. 1).

### Analysis of ROC curves

**Fig. 2:**
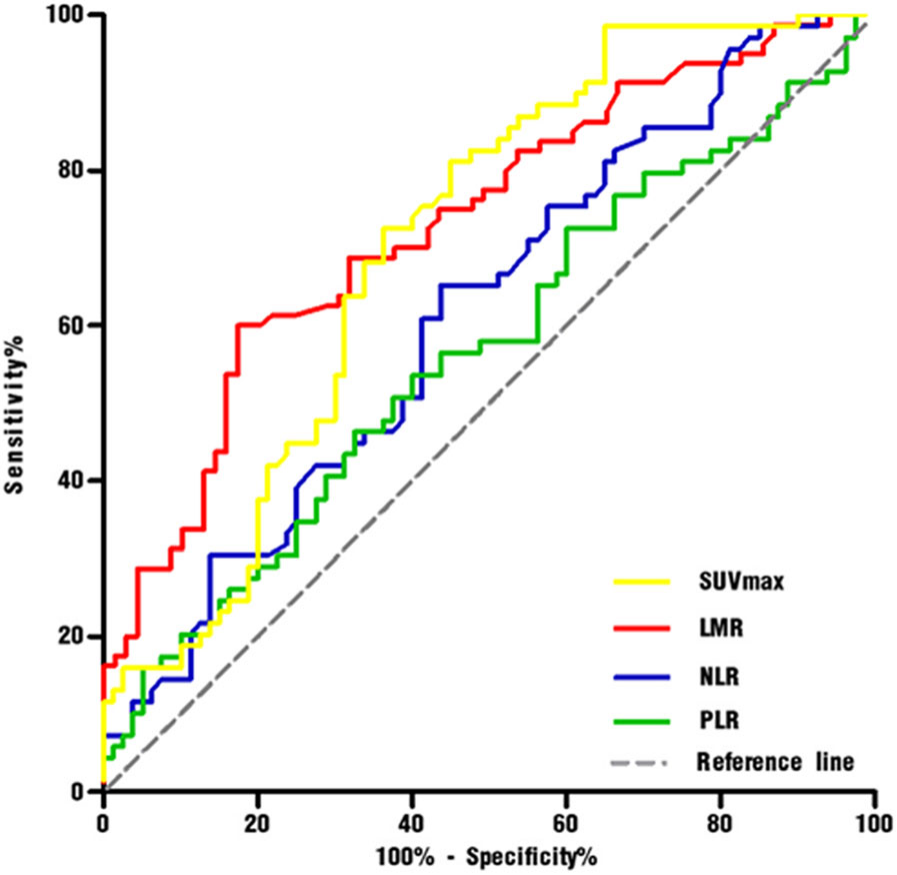
Receiver operating characteristic curves for SUVmax and hematological markers

The AUC of SUVmax for identifying responders was 0.705, with an optimal cut-off value of 11.6 (sensitivity 72.5%, specificity 63.7%) (Fig. 2). The AUC of LMR, NLR and PLR was 0.732, 0.615 and 0.566, respectively. According to the Delong’s test, the AUC of LMR was higher than that of NLR (ΔAUC = 0.117, *p* = 0.004) and of PLR (ΔAUC = 0.165, *p* = 0.001). The optimal cut-off values of LMR, NLR, and PLR were 3.73 (sensitivity 60%, specificity 82.6%), 2.67 (sensitivity 65.2%, specificity 56.2%), and 164 (sensitivity 46.4%, specificity 67.5%), respectively (Fig. 2). Patients were divided into high and low groups based on these optimal cut-off values.

### Univariable and multivariable analyses of tumor response

Of the 149 NSCLC patients, CR, PR, SD and PD occurred in 3 (2.0%), 77 (51.7%), 61 (40.9%) and 8 (5.4%) cases, respectively. The overall ORR was 53.7% (80/149).

In univariable analyses, those with SUVmax values ≤11.6 had a higher ORR than did those with SUVmax > 11.6 (72.5% vs 37.5%, *p* < 0.001), whereas those with LMR > 3.73 had a higher ORR than did those with LMR ≤ 3.73 (80% vs 36%, *p* < 0.001). In addition to high SUVmax and low LMR, old age (*p* = 0.042), high ECOG PS (*p* = 0.001), advanced clinical stage (*p* = 0.004), increased CEA level (p = 0.042), high WBC (*p* = 0.003) and NLR (*p* = 0.009) were also associated with poor ORRs.

In multivariable analyses, the SUVmax (odds ratio [OR]: 0.217; 95% confidence interval [CI]: 0.090–0.525; p = 0.001), LMR (OR: 0.167; 95% CI: 0.062–0.454; *p* < 0.001), and clinical stage (OR: 0.283; 95% CI: 0.112–0.718; *p* = 0.008) were demonstrated as independent predictors of treatment response (Table 2).

**Table 2:** Univariable and multivariable analyses of the first-line response to chemotherapy

Variables	Tumor response			Univariable Analysis		Multivariable analysis	
	CR + PR	SD + PD	ORR	OR (95% CI)	***p*** value	OR (95% CI)	***p*** value
Age (years)					0.042*		0.251
≤ 65	57	38	60.0%	Ref.		Ref.	
>65	23	31	42.6%	0.495 (0.251–0.974)		0.593 (0.243–1.447)	
Sex					0.177		
Male	56	55	50.5%	0.594 (0.279–1.266)			
Female	24	14	63.2%	Ref.			
Smoking					0.550		
Never	34	26	56.7%	Ref.			
Ever	46	43	51.7%	0.818 (0.423–1.580)			
ECOG PS					0.001**		0.302
0	49	24	67.1%	Ref.		Ref.	
1	31	45	40.8%	0.337 (0.173–0.659)		0.646 (0.282–1.481)	
Lesion type					0.344		
Peripheral	41	30	57.7%	Ref.			
Central	39	39	50.0%	0.732 (0.383–1.398)			
Histological type					0.857		
Adenocarcinoma	51	43	54.3%	Ref.			
Squamous	29	26	52.7%	0.940 (0.483–1.832)			
Clinical stage					0.004**		0.008**
IIIB	46	23	66.7%	Ref.		Ref.	
IV	34	46	42.5%	0.370 (0.189–0.721)		0.283 (0.112–0.718)	
CEA					0.042*		0.170
Normal	57	38	60.0%	Ref.		Ref.	
Increased	23	31	42.6%	0.495 (0.251–0.974)		0.539 (0.223–1.303)	
Albumin					0.093		
Decreased	22	28	44.0%	0.555 (0.279–1.104)			
Normal	58	41	58.6%	Ref.			
WBC(×10^9^/L)					0.003**		0.424
≤ 7.8	53	29	64.6%	Ref.		Ref.	
>7.8	27	40	40.3%	0.369 (0.190–0.719)		0.710 (0.307–1.644)	
LMR					< 0.001***		< 0.001***
≤ 3.73	32	57	36.0%	0.140 (0.065–0.302)		0.167 (0.062–0.454)	
>3.73	48	12	80.0%	Ref.		Ref.	
NLR					0.009**		0.737
≤ 2.67	45	24	65.2%	Ref.		Ref.	
>2.67	35	45	43.8%	0.415 (0.214–0.806)		1.176 (0.456–3.032)	
PLR					0.084		
≤ 164	54	37	59.3%	Ref.			
>164	26	32	44.8%	0.557 (0.286–1.083)			
SUVmax					< 0.001***		0.001**
≤ 11.6	50	19	72.5%	Ref.		Ref.	
>11.6	30	50	37.5%	0.228 (0.114–0.457)		0.217 (0.090–0.525)	

*ECOG PS* Eastern Cooperative Oncology Group Performance Status, *CEA* carcinoembryonic antigen, *WBC* white blood cell, *LMR* lymphocyte-monocyte ratio, *NLR* neutrophil-lymphocyte ratio, *PLR* platelet-lymphocyte ratio, *SUVmax* maximum standardized uptake value, *CR* complete response, *PR* partial response, *SD* stable disease, *PD* progressive disease, *ORR* objective response rate, *OR* odds ratio, *CI* confidence interval. *, *p* < 0.05; **, *p* < 0.01; ***, *p* < 0.001

### Univariable and multivariable analyses of PFS and OS

**Fig. 3:**
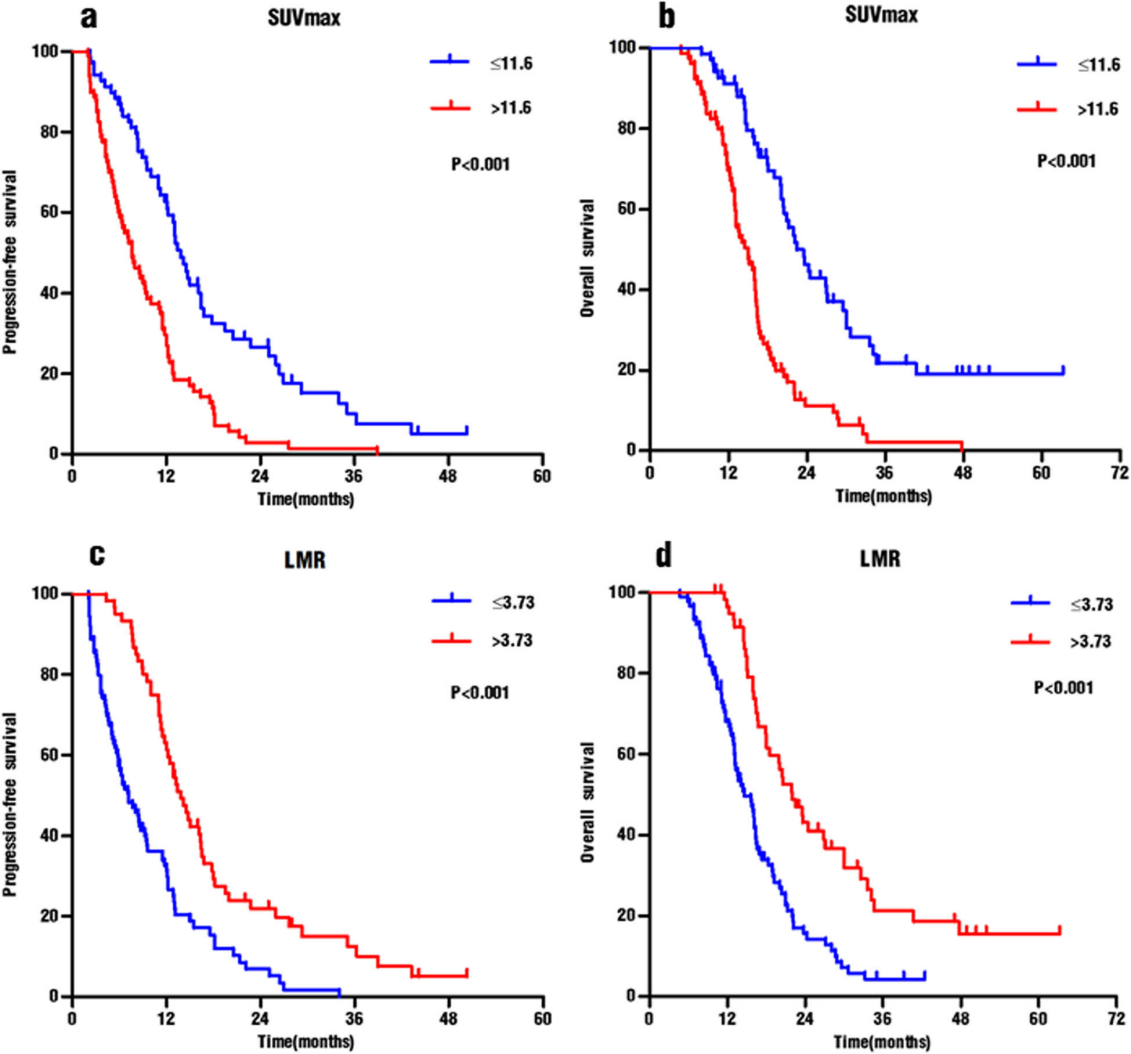
Kaplan–Meier analyses of 149 patients with stage IIIB-IV NSCLC. **a** Progression-free survival curves according to SUVmax. **b** Overall survival curves according to SUVmax. **c** Progression-free survival curves according to LMR. **d** Overall survival curves according to LMR

KM curves analysis showed that patients with a high SUVmax (> 11.6) exhibited significantly shorter PFS and OS than did those with low SUVmax (median PFS: 7.6 vs 13.4 months, *p* < 0.001; median OS: 14.5 vs 22.5 months, p < 0.001) (Fig. 3a-b). Similarly, the PFS and OS of patients with low LMR (≤3.73) were significantly lower than those with high LMR (median PFS: 7.1 vs 13.4 months, *p* < 0.001; Median OS: 14.4 vs 21.9 months, *p* < 0.001) (Fig. 3c-d).

In the univariable analysis, age (PFS, *p* = 0.015; OS, *p* = 0.008), ECOG PS (PFS, *p* = 0.007; OS, *p* = 0.019), clinical stage (PFS, *p* = 0.017; OS, *p* = 0.045), first-line response (PFS, *p* < 0.001; OS, *p* < 0.001), LMR (PFS, *p* < 0.001; OS, *p* < 0.001) and SUVmax (PFS, *p* < 0.001; OS, *p* < 0.001) were significantly correlated with PFS and OS. Albumin level also correlated with PFS (*p* = 0.023) (Table 3).

**Table 3:** Univariable analyses of PFS and OS

Variables	PFS			OS		
	HR	95% CI	***p*** value	HR	95% CI	***p*** value
Age (years)			0.015*			0.008**
≤ 65	Ref.			Ref.		
>65	1.567	1.093–2.248		1.666	1.142–2.429	
Sex			0.174			0.365
Male	1.320	0.885–1.968		1.216	0.796–1.859	
Female	Ref.			Ref.		
Smoking			0.391			0.717
Never	Ref.			Ref.		
Ever	1.168	0.819–1.665		1.070	0.741–1.547	
ECOG PS			0.007**			0.019*
0	Ref.			Ref.		
1	1.610	1.136–2.283		1.548	1.073–2.234	
Lesion type			0.086			0.443
Peripheral	Ref.			Ref.		
Central	1.357	0.958–1.922		1.152	0.802–1.655	
Histological type			0.835			0.816
Adenocarcinoma	Ref.			Ref.		
Squamous	1.038	0.728–1.482		0.957	0.661–1.386	
Clinical stage			0.017*			0.045*
IIIB	Ref.			Ref.		
IV	1.529	1.078–2.168		1.450	1.008–2.086	
First-line response			< 0.001***			< 0.001***
CR/PR	Ref.			Ref.		
SD/PD	2.725	1.905–3.898		2.966	2.044–4.306	
CEA			0.280			0.719
Normal	1.219	0.851–1.746		1.071	0.737–1.557	
Increased	Ref.			Ref.		
Albumin			0.023*			0.140
Decreased	1.521	1.059–2.183		1.323	0.913–1.918	
Normal	Ref.			Ref.		
WBC			0.098			0.158
≤ 7.8	Ref.			Ref.		
>7.8	1.337	0.948–1.887		1.297	0.904–1.860	
LMR			< 0.001***			< 0.001***
≤ 3.73	2.373	1.643–3.429		2.437	1.659–3.579	
>3.73	Ref.			Ref.		
NLR			0.064			0.067
≤ 2.67	Ref.			Ref.		
>2.67	1.395	0.981–1.984		1.403	0.977–2.017	
PLR			0.107			0.101
≤ 164	Ref.			Ref.		
>164	1.342	0.939–1.918		1.361	0.942–1.968	
SUVmax			< 0.001***			< 0.001***
≤ 11.6	Ref.			Ref.		
>11.6	2.400	1.674–3.440		2.976	2.024–4.375	
LMR_SUV			< 0.001***			< 0.001***
Score 0	Ref.			Ref.		
Score 1	2.228	1.383–3.590	0.001**	2.426	1.441–4.085	0.001**
Score 2	4.449	2.719–7.281	< 0.001***	5.361	3.172–9.058	< 0.001***

*PFS* progression-free survival, *OS* overall survival, *ECOG PS* Eastern Cooperative Oncology Group Performance Status, *CR* complete response, *PR* partial response, *SD* stable disease, *PD* progressive disease, *CEA* carcinoembryonic antigen, *WBC* white blood cell, *LMR* lymphocyte-monocyte ratio, *NLR* neutrophil-lymphocyte ratio, *PLR* platelet-lymphocyte ratio, *SUVmax* maximum standardized uptake value, *HR* hazard ratio, *CI* confidence intervals. *, *p* < 0.05; **, *p* < 0.01; ***, *p* < 0.001

In the multivariable analysis, the SUVmax was independently correlated with both PFS (hazard ratio [HR]: 2.110; 95%CI: 1.400–3.179; p < 0.001) and OS (HR: 2.760; 95%CI: 1.789–4.257; *p* < 0.001). Furthermore, LMR was also independently associated with PFS (HR: 1.602; 95%CI: 1.060–2.420; *p* = 0.025) and OS (HR: 1.621; 95%CI: 1.042–2.521; *p* = 0.032). Other independent prognostic factors for PFS and OS included clinical stage (PFS, *p* = 0.003; OS, *p* = 0.002) and first-line response (PFS, *p* = 0.037; OS, *p* = 0.011) (Table 4, Model 1).

**Table 4:** Multivariable analyses of PFS and OS (Model 1)

Variables	PFS			OS		
	HR	95% CI	***p*** value	HR	95% CI	***p*** value
Age (years)			0.443			0.973
≤ 65	Ref.			Ref.		
>65	1.177	0.776–1.787		0.992	0.634–1.553	
ECOG PS			0.443			0.350
0	Ref.			Ref.		
1	1.164	0.790–1.714		1.214	0.808–1.823	
Clinical stage			0.003**			0.002**
IIIB	Ref.			Ref.		
IV	1.807	1.229–2.658		1.880	1.260–2.806	
First-line response			0.037*			0.011*
CR/PR	Ref.			Ref.		
SD/PD	1.572	1.027–2.407		1.799	1.143–2.831	
Albumin			0.089			
Decreased	1.377	0.952–1.991				
Normal	Ref.					
LMR			0.025*			0.032*
≤ 3.73	1.602	1.060–2.420		1.621	1.042–2.521	
>3.73	Ref.			Ref.		
SUVmax			< 0.001***			< 0.001***
≤ 11.6	Ref.			Ref.		
>11.6	2.110	1.400–3.179		2.760	1.789–4.257	

*PFS* progression-free survival, *OS* overall survival, *ECOG PS* Eastern Cooperative Oncology Group Performance Status, *CR* complete response, *PR* partial response, *SD* stable disease, *PD* progressive disease, *LMR* lymphocyte-monocyte ratio, *SUVmax* maximum standardized uptake value, *HR* hazard ratio, *CI* confidence intervals. *, *p* < 0.05; **, *p* < 0.01; ***, *p* < 0.001

### The value of the LMR_SUV score in predicting treatment response and survival

To further discriminate patients with different outcomes, a novel scoring system termed the SUV_LMR score that includes SUVmax and LMR was constructed. The score was categorized as follows: score 0, patients with low SUVmax (≤11.6) and high LMR (> 3.73); score 2, patients with high SUVmax (> 11.6) and low LMR (≤3.73); and score 1, all remaining patients.

First, we evaluated the differences in treatment response among the three subgroups. The patients with a score of 0 had the highest ORR of 88.9%, whereas patients with a score of 2 had the lowest ORR of only 25%. In addition, the ORR of patients with a score of 1 was intermediate, at 59.6%. More importantly, significant inter-group differences in the ORRs were observed (0 vs 1, *p* = 0.002, 1 vs 2, *p* < 0.001; and 0 vs 2, *p* < 0.001) (Table 5).

**Table 5:** Chemotherapeutic response according to LMR_SUV scores

SUV_LMR score	PR + CR	SD + PD	ORR	Compared	***p*** value	***p*** value
Score 0 (*n* = 36)	32	4	88.9%	Score 1	0.002**	< 0.001***
				Score 2	< 0.001***	
Score 1 (*n* = 57)	34	23	59.6%	Score 2	< 0.001***	
Score 2 (*n* = 56)	14	42	25.0%			

*SUV* standardized uptake value, *LMR* lymphocyte-monocyte ratio, *CR* complete response, *PR* partial response, *SD* stable disease, *PD* progressive disease, *ORR* objective response rate. *, *p* < 0.05; **, *p* < 0.01; ***, *p* < 0.001

**Fig. 4:**
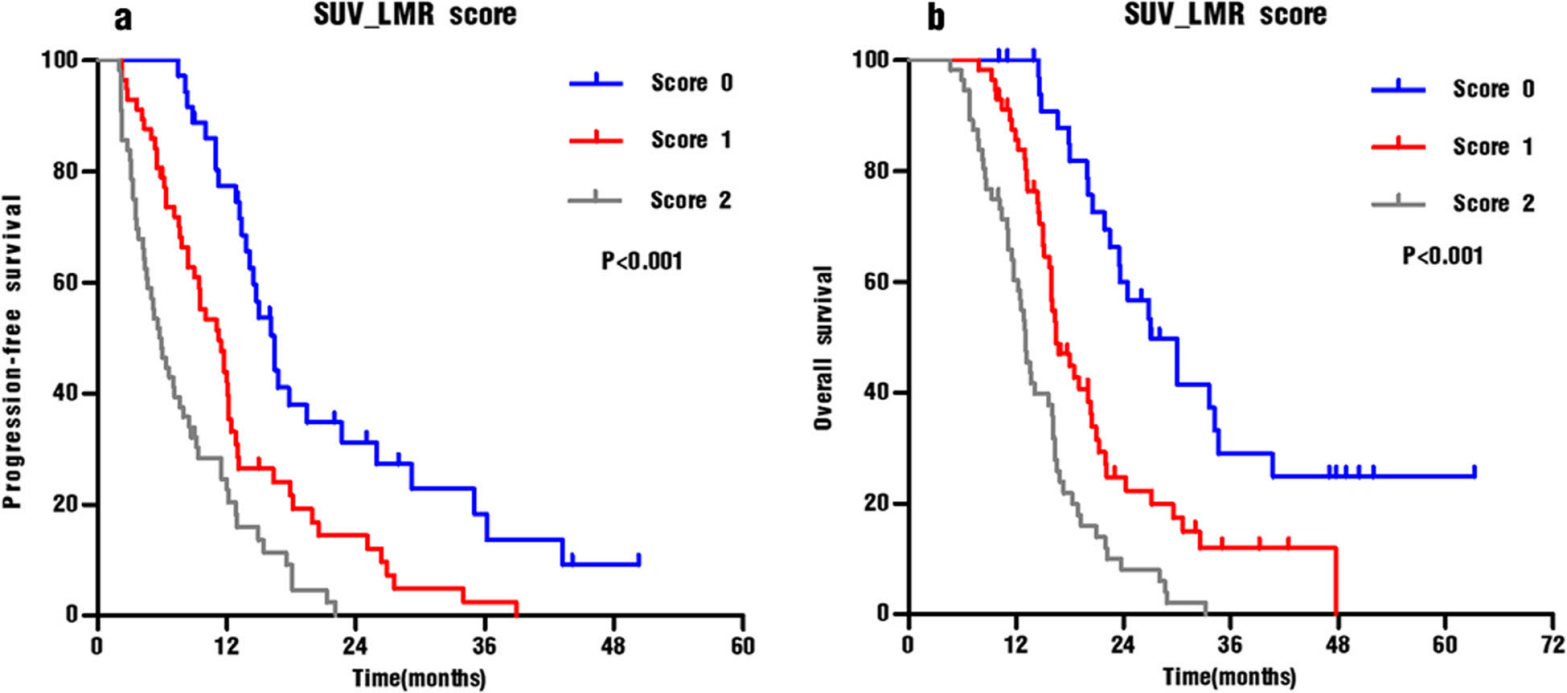
Kaplan–Meier analyses for progression-free survival **a** and overall survival **b** of IIIB-IV NSCLC patients according to the SUV_LMR score (0,1 and 2)

Moreover, we examined the prognostic value of the SUV_LMR score. The median PFS for patients with scores of 0, 1, and 2 was 16.1, 11.1, and 5.8 months, respectively (Fig. 4a, *p* < 0.001). Differences in PFS according to the SUV_LMR scores were significant (0 vs 1, *p* < 0.001; 1 vs 2, *p* = 0.001;0 vs 2, *p* < 0.001). Similar results were obtained for OS. The median OS for patients with scores of 0, 1, and 2 was 26.9, 16.4, and 13.0 months, respectively (Fig. 4b, *p* < 0.001). The OS stratifications according to the SUV_LMR scores were also significant (0 vs 1, *p* < 0.001; 1 vs 2, *p* < 0.001; 0 vs 2, *p* < 0.001).

To further evaluate the prognostic value of the SUV_LMR score whilst avoiding the effect of SUVmax and LMR on the SUV_LMR score, another multivariable Cox regression model (Model 2) was constructed. The results demonstrated that the SUV_LMR score was independently associated with PFS (*p* < 0.001) and OS (*p* < 0.001) (Table 6, Model 2). The patients with a score of 0 exhibited the longest PFS and OS of the subgroups. Patients with a score of 1 (HR: 2.017; 95%CI: 1.233–3.300; *p* = 0.005) and score of 2 (HR: 3.421; 95%CI: 1.903–6.148; p < 0.001) exhibited significantly decreased PFS compared with that of patients with a score of 0. Similarly, patients with a score of 1 (HR: 2.177; 95%CI: 1.273–3.722; *p* = 0.004) and score of 2 (HR: 4.573; 95% CI: 2.441–8.569; p < 0.001) also showed significantly worse OS compared with that of patients with a score of 0.

**Table 6:** Multivariable analyses of PFS and OS (Model 2)

Variables	PFS			OS		
	HR	95% CI	p value	HR	95% CI	***p*** value
Age (years)			0.379			0.975
≤ 65	Ref.			Ref.		
>65	1.204	0.796–1.824		0.993	0.640–1.540	
ECOG PS			0.487			0.483
0	Ref.			Ref.		
1	1.147	0.780–1.686		1.154	0.773–1.724	
Clinical stage			0.004**			0.004**
IIIB	Ref.			Ref.		
IV	1.752	1.190–2.578		1.802	1.207–2.690	
First-line response			0.035*			0.015*
CR/PR	Ref.			Ref.		
SD/PD	1.576	1.033–2.406		1.754	1.117–2.753	
Albumin			0.097			
Decreased	1.367	0.945–1.978				
Normal	Ref.					
LMR_SUV			< 0.001***			< 0.001***
Score 0	Ref.			Ref.		
Score 1	2.017	1.233–3.300	0.005**	2.177	1.273–3.722	0.004**
Score 2	3.421	1.903–6.148	< 0.001***	4.573	2.441–8.569	< 0.001***

*PFS* progression-free survival, *OS* overall survival, *ECOG PS* Eastern Cooperative Oncology Group Performance Status, *CR* complete response, *PR* partial response, *SD* stable disease, *PD* progressive disease, *LMR* lymphocyte-monocyte ratio, *SUVmax* maximum standardized uptake value, *HR* hazard ratio, *CI* confidence intervals. *, *p* < 0.05; **, *p* < 0.01; ***, *p* < 0.001

## Discussion

Our retrospective study revealed that pre-treatment SUVmax and LMR were independent factors for predicting treatment response and prognosis in stage IIIB-IV NSCLC patients receiving chemotherapy. More importantly, an innovative scoring system based on SUVmax and LMR was constructed that can serve as an accurate and effective tool for predicting chemotherapeutic response and prognosis.

SUVmax is one of the most important parameters derived from PET that can accurately measure the metabolic activity of tumors and provide valuable prognostic information. Although the cellular and molecular mechanisms are not well known, some studies have reported that SUVmax is closely associated with biological factors that influence cancer proliferation and progression in NSCLC, such as Ki-67 [27, 28] and VEGF [28]. A large prospective study by Vesselle et al. [27] investigated the correlation between Ki-67 expression and tumor FDG uptake in 178 patients with NSCLC. The results showed that SUVmax was positively correlated with Ki-67 scores in tumor tissue, and it is well known that the overexpression of Ki-67 in tumors indicates active proliferative activity and aggressive biological behaviour. In another study on NSCLC, Takenaka et al. [28] assessed the relationship between SUVmax and intratumoral expression of VEGF and demonstrated that SUVmax was significantly higher in patients with high expression of VEGF, which plays an important role in tumor angiogenesis, invasion, and metastasis. Above studies revealed that SUVmax could reflect tumor proliferation and angiogenesis, which is related to prognosis. In NSCLC, many studies have demonstrated the prognostic significance of SUVmax [10, 29]. A meta-analysis by Berghmans et al. [29] encompassing 13 studies highlighted tumor SUVmax values as a prognostic factor of NSCLC. In another retrospective study of 315 NSCLC patients, Cerfolio et al. [10] found that patients with SUVmax ≥10 were more likely to have cancer recurrence and shorter survival compared with those of patients with low SUVmax. More importantly, they also concluded that SUVmax was a more powerful independent prognostic factor than TNM stage [10]. Consistent with these findings, we also observed that high SUVmax values (> 11.6) of primary tumor is independently associated with poor PFS and OS. In addition, we also demonstrated that a high SUVmax was a significant predictive marker of poor chemotherapeutic response. The mechanism may be related to the overexpression of p53 in tumor cells, as a previous study confirmed the positive correlation between SUVmax and p53 expression [30], and p53 overexpression has been proved to be significantly correlated with chemotherapy resistance in lung cancer [31]. This may be an underlying mechanism to explain why the high SUVmax is associated with poor chemotherapeutic effects.

Accumulating evidence has demonstrated that systemic inflammation plays a key role in tumorigenesis, progression and metastasis [17]. LMR, one of the simple markers of systemic inflammatory response, has been shown to correlate with clinical outcomes of NSCLC [25, 26]. In a retrospective assessment of 107 patients with advanced lung squamous cell carcinoma who received chemotherapy, Minami et al. showed that a low LMR (< 2.07) could independently predict a poor OS, while NLR could not [25]. In terms of short-term efficacy, their results showed that the high LMR group exhibited a higher response rate to chemotherapy than did the low LMR group (25). Lin et al. also reported that high LMR values (> 4.56) were predictive of a longer PFS and OS in 370 metastatic NSCLC patients receiving chemotherapy [26]. Our data were consistent with these findings, as a low pre-treatment LMR (≤3.73) was a significant predictor of unfavourable chemotherapeutic responses and a poor PFS and OS. The exact mechanisms of these associations currently remain unclear, but there are some hypotheses on this issue. On the one hand, it is well known that lymphocytes, especially cytotoxic T lymphocytes, play a critical role in the antitumor immune response by inducing cytotoxic cell death and suppressing tumor cell proliferation and invasion [32]. A previous study has demonstrated that a low lymphocyte count was associated with a poor DFS in NSCLC patients [33]. On the other hand, circulating monocytes can differentiate into tumor-associated macrophages (TAMs) in the tumor microenvironment, and growing evidence suggests that TAMs promote cancer initiation, progression and metastasis by inducing mutagenesis, stimulating angiogenesis, and suppressing anti-tumor immunity [16–18]. All the above theories suggested that the LMR can reflect the balance between the anti-tumor immunity of the lymphatic system and the unfavourable tumor-promoting effects of monocytes. A low LMR indicates a relatively decreased lymphocyte count and an increased monocyte count, so it may predict a poor clinical outcome.

To summarize, SUVmax represents the local metabolic status of primary tumor, and LMR reflects the host’s systemic inflammatory response. The comprehensive evaluation of these two factors may be more accurate and effective at predicting the chemotherapeutic response and prognosis. Interestingly, we demonstrated a weak but significant negative correlation between SUVmax and LMR in this study. A similar correlation between SUVmax and hematological parameters was observed in colorectal cancer [34], NSCLC [35] and breast cancer [36]. In a study of colorectal cancer, Xu et al. [34] demonstrated that SUVmax was significantly correlated with LMR and NLR. In addition, in other studies on NSCLC and breast cancer, Jeong et al. [35] and Fujii et al. [36] also demonstrated the correlation between SUVmax and NLR. The results of previous studies may offer some explanations for this correlation. One potential opinion was that inflammatory cells infiltrating the tumor microenvironment, such as neutrophils and macrophages, also consumed FDG, resulting in increased FDG uptake throughout the tumor [37]. Another possible explanation may be related to inflammation-induced angiogenesis. Inflammation promotes hypoxia in the tumor microenvironment and induces angiogenesis by stimulating VEGF secretion [38]. Then, the uptake of FDG will increase significantly during tumor angiogenesis [39]. These analyses shed new insight into the relationship between tumor metabolic activity and the host’s inflammatory response process. Therefore, in present study, we established a scoring system based on the SUVmax and LMR, and demonstrated its value to predict PFS and OS, highlighting its prognostic potential. Patients with baseline SUVmax ≤11.6 along with LMR > 3.73 (score 0) exhibited the longest PFS and OS of the three groups, whereas patients with both pre-treatment SUVmax > 11.6 and LMR ≤3.73 (score 2) showed the worst treatment outcome, with significantly poorer OS and PFS. Another highlight of this study was the association between the SUV_LMR score and first-line chemotherapeutic response. Patients with a score of 0 had the highest ORR among the subgroups, while patients with a score of 2 had the worst ORR. The SUV_LMR score therefore has the potential to predict treatment response and prognosis and may be helpful in selecting appropriate treatment strategies for advanced patients. Patients with a score of 0 appeared to be more sensitive to chemotherapy, so the current platinum-based doublet chemotherapy may be the optimal strategy for them; thus, for these patients, chemotherapy should be implemented as soon as possible. However, for patients with a score of 2, they seemed to be relatively insensitive to chemotherapy, and their prognosis was extremely poor. Therefore, alternative strategies should be considered for these patients, such as molecularly targeted therapies and immunotherapies.

Some study limitations should be discussed. The study was retrospective and from a single centre, and the number of patients was small. Secondly, selection criteria were limited to stage IIIB-IV NSCLC, and the prognostic impact of the SUV_LMR scores may be differ in those at early disease stages. Finally, the predictive performance of the SUV_LMR score needs further validation in larger prospective studies containing more samples.

## Conclusions

Pre-treatment SUVmax and LMR were independent predictive factors of clinical tumor response and prognosis in stage IIIB-IV NSCLC patients who were treated with first-line chemotherapy. More importantly, the SUV_LMR score, which is based on primary tumor metabolic activity and the systemic inflammatory response, provides a promising tool to predict chemosensitivity, recurrence and survival of advanced NSCLC.

## Data Availability

The datasets used and/or analysed during the current study are available from the corresponding author on reasonable request.

## Abbreviations

NSCLC: Non-small cell lung cancer
TNM: Tumor, node, metastasis
FDG-PET/CT: Fluorodeoxyglucose positron emission tomography/computed tomography
SUVmax: Maximum standardized uptake value
LMR: Lymphocyte-monocyte ratio
NLR: Neutrophil-lymphocyte ratio
PLR: Platelet- lymphocyte ratio
ECOG PS: Eastern cooperative oncology group performance status
WBC: White blood cell
ORR: Objective response rates
CR: Complete response
PR: Partial response
SD: Stable disease
PD: Progressive disease
OS: Overall survival
PFS: Progression-free survival
ROC: Receiver-operating characteristic
AUC: Area under the curves
VEGF: Vascular endothelial growth factor
